# Improving the proof of “Privacy-preserving attribute-keyword based data publish-subscribe service on cloud platforms”

**DOI:** 10.1371/journal.pone.0212761

**Published:** 2019-02-25

**Authors:** Shangping Wang, Qian Zhang, Yaling Zhang, Jin Sun, Juanjuan Chen, Xiaoqing Sun

**Affiliations:** 1 School of Science, Xi’an University of Technology, Xi’an, Shaanxi, China; 2 School of Computer Science and Engineering, Xi’an University of Technology, Xi’an, Shaanxi, China; University of Nevada, UNITED STATES

## Abstract

Most recently, Kan Yang et al. proposed an attribute-keyword based encryption scheme for data publish-subscribe service(AKPS), which is highly useful for cloud storage scenario. Unfortunately, we discover that there is a flaw in the security proof of indistinguishability of the tag and trapdoor against chosen keyword attack under the Bilinear Diffie-Hellman (BDH) assumption. As the security proof is a key component for a cryptographic scheme, based on the Decisional Diffie-Hellman (DDH) assumption, we improve the security proof method and give a new security proof of the AKPS scheme for indistinguishability of the tag and trapdoor in our proposal, which is more rigorous than the original one. Furthermore, we also demonstrate that the AKPS scheme is secure against data Replayable Chosen Ciphertext Attack (RCCA).

## I. Introduction

Data publish-subscribe system [1, 2] is an appropriate mode for data users to receive data for interest. Cloud server, owing to considerable resources on storage and calculation, has been proven to be the most applicable platform for this service [3–5].

To realize fine-grained access control of data on cloud storage, the concept of the attribute-based encryption (ABE) was proposed. Generally ABE can be divided into two categories: Ciphertext- Policy Attribute-based Encryption (CP-ABE) [6, 7] and Key-Policy Attribute-based Encryption (KP-ABE) [8], both are intended for one-to-many access mode. Attribute-based encryption is an extension of public-key cryptography and identity-based cryptography. Compared with traditional cryptography, attribute-based encryption provides a more flexible encryption and decryption relationship. For example, in an attribute-based encryption mechanism, both the ciphertext and the secret key are associated with a set of attributes, and the data owner can specify an encryption policy consisting of attributes, and the resulting ciphertext can be decrypted only by the data user whose attributes satisfies the encryption policy. The non-interactive access control with fine-grained can be realized effectively by attribute-based encryption, which greatly enriches the flexibility of encryption policy and the description of user's rights. Due to its high efficiency, dynamic, flexibility and privacy, attribute-based encryption has a good application foreground in distributed file management, third party data storage and pay-TV system. With the development and popularization of cloud computing technology, more and more enterprises and users are outsourcing their data to cloud service providers, which providing a good way to protect data security and privacy by applying attribute-based encryption.

In [9, 10], a Dual-policy ABE was created to achieve CP-ABE and KP-ABE simultaneously, and it was appropriate for data publish-subscribe system as it enables the publishers and the subscribers to define individual policy according to the feature. A publish-subscribe system should meet the following requirements. (i) The publisher publishes data and specifies which subscribers can access his/her data and the subscriber may specify the data he/she is interested in; (ii) Allow subscribers to perform multiple keyword search; (iii) Search queries that allow multiple users to retrieve data. Considering data privacy, the keyword privacy in the tag and trapdoor and the decryption overhead from the subscriber, Kan Yang proposed a privacy-preserving attribute-keyword based data publish-subscribe (AKPS) scheme [1]. Firstly, the AKPS scheme implemented the access policy and subscription policy respectively by using dual policy attribute-based encryption, which could make the subscriber's attributes satisfy the publisher's access policy and the data released by the publisher also satisfied subscription policy specified by the subscriber. In this way, the AKPS scheme could achieve the fine-grained two-way access control. Secondly, by using the concept of attribute-keyword, the subscriber's subscription policy was constructed, which was designed to avoid the leaking of keywords information and realized the multi-keyword search and the expression of subscription policy. Thirdly, attribute-keyword based data publish-subscribe scheme supported multiple publishers and multiple subscribers, and could be used to data sharing with access control in publish-subscribe system on cloud platforms. Finally, the decryption overhead was transferred from the subscribers’ devices to the cloud supporter to reduce the subscriber’s computational burden by using outsourcing decryption technology [11–13], which was a practical tool for lightening the computational load on the subscriber side in reality result from that mobile devices have become primary computing device for many users and enterprises.

Unfortunately, we discovered that the security proof of the AKPS scheme [1] was not enough rigorous and adequate after carefully researching it. According to the security proof of the AKPS scheme[1], we find with random guessing, the adversary can solve the Bilinear Diffie-Hellman (BDH) problem with a probability of 1/2, and their security proof actually has nothing to do with the adversary’s attacking of the scheme (the detail is refer to section IV). Through analyzing the security of the original AKPS scheme [1], we also find it is hard to apply the BDH assumption to the security proof of the AKPS scheme. Therefore in order to prove the security of the chosen keyword attack for indistinguishability of the tag and the trapdoor for the AKPS scheme, we carry out a detailed analysis of the AKPS scheme and study the basic steps of the provable security method. Through careful analysis and research, we discover that the Decisional Diffie-Hellman (DDH) assumption can be used to prove the security of the AKPS scheme.

In view of this, we improve the proof method and design a new security proof of the AKPS scheme for indistinguishability of the tag and trapdoor based on the DDH assumption, our new security proof is more rigorous than their original proof. In addition, Chosen Ciphertext Attack (CCA) security [12, 14] was regarded as the appropriate security notion for encryption schemes used as components in general protocols and applications. Whereas, there exists a weaker secure notion called Replayable Chosen Ciphertext Attack (RCCA) [15] security than the CCA security, and has been proven to be sufficient for most actual intention. In this paper we prove that the data security of the AKPS scheme is of RCCA security, which is not presented in [1].

## Our Contributions

(1) We show that the security proof of the AKPS scheme [1] is not enough rigorous and adequate, and we give a detail analysis about it in section IV.

(2) We improve the security proof method and present a new security proof of the AKPS scheme for indistinguishability of the tag and trapdoor based on the DDH assumption.

(3) Using the conclusion that the Waters’s scheme in [6] is the selectively CPA-secure, we can prove that the AKPS scheme realizes data security against replayable chosen ciphertext attack (RCCA).

**Fig 1:**
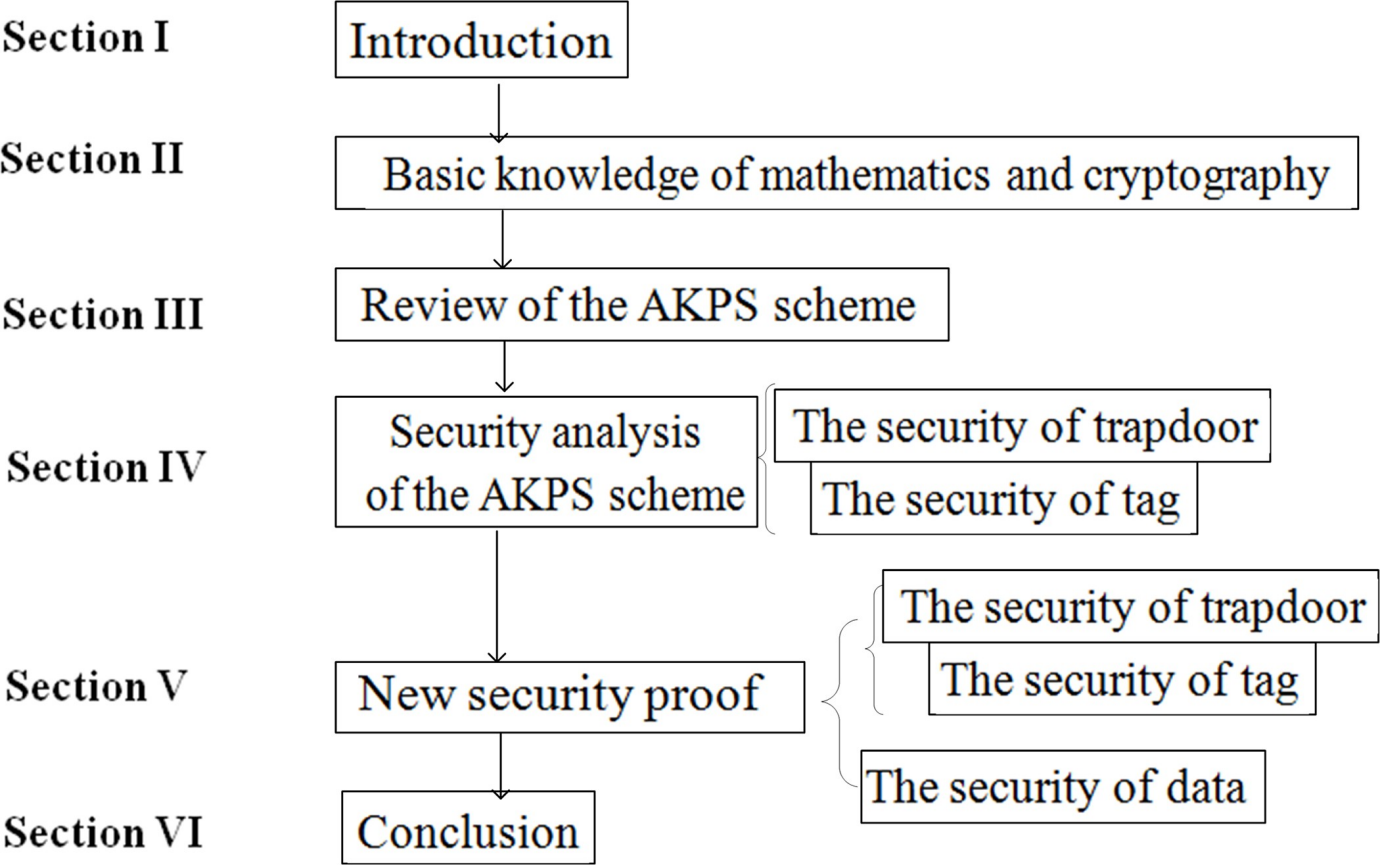
Flaw-chart in the system.

In order to make the overall layout of the system clearer, the flaw-chart of the system is shown in Fig 1.

## II. Preliminaries

In this section, we present the basics of mathematics and cryptography required in the scheme, including the deterministic assumptions used in the proof, system model of the AKPS scheme and the security definition of the AKPS scheme.

### A. Decisional Diffie-Hellman (DDH) assumption

**Definition 1** (DDH [16]).Let *x*,*y*,*z*∈*Z*_*p*_ be chosen at random and *g* be a generator of *G*. The Decisional DH assumption is that there is no probabilistic polynomial time algorithm *P* can distinguish the tuple (*A* = *g*^*x*^,*B* = *g*^*y*^,*C* = *g*^*xy*^) from the tuple (*A* = *g*^*x*^,*B* = *g*^*y*^,*C* = *g*^*z*^) with more than a negligible advantage *ε*. The advantage of *P* is defined as |*Pr*[(*A*,*B*,*g*^*xy*^) = 0]−*Pr*[*A*,*B*,*g*^*z*^] = 0| = *ε*.

### B. Decision *q*-parallel Bilinear Diffie-Hellman Exponent (BDHE) assumption

**Definition 2** (Decision *q*-parallel BDHE [11]). Let *a*,*s*,*b*_1_,⋯,*b*_*q*_∈*Z*_*p*_ be chosen randomly and *g* be a generator of *G*. If an adversary is given by

$$\vec{y}=(g,g^{s},g^{\frac{1}{z}},g^{\frac{a}{z}},\cdots ,g^{\left(\frac{a^{q}}{z}\right)},g^{a},\cdots ,g^{\left(a^{q}\right)},,g^{\left(a^{q+2}\right)},\cdots ,g^{\left(a^{2q}\right)},$$

$$\forall 1\leq j\leq q:\;g^{s∙b_{j}},g^{\frac{a}{b_{j}}},\cdots ,g^{\left(\frac{a^{q}}{b_{j}}\right)},\cdots ,g^{\left(a^{q}\right)},,g^{\left(\frac{a^{q+2}}{b_{j}}\right)},\cdots ,g^{\left(\frac{a^{2q}}{b_{j}}\right)},$$

∀1≤j,k≤q,k≠j:ga∙s∙bkbj,⋯,g(aq∙s∙bkbj),

It must be hard to distinguish a valid tuple ${e(g,g)}^{a^{q+1}s}\in G_{T}$ from a random element *R* in *G*_*T*_. An algorithm B has advantage *ε* in solving *q*-parallel BDHE in *G* if ${\left|Pr[B(\vec{y},T=e(g,g\right)}^{a^{q+1}s}]=0-Pr[B(\vec{y},T=R]=0|\geq \varepsilon$.

### C. System model of AKPS

At the beginning, we give some notations that will appear in the AKPS scheme as shown in Table 1.

**Table 1:** Notations.

Notation	Description
*msk*	master key of the authority
*pk*	public parameter of the system
*sk*_*sub*_	private key of subscriber
*sk*_*pub*_	private key of publisher
*Td*_*sub*_	keyword trapdoor of subscriber
*pdk*_*sub*_	pre-decryption key of subscriber
*dk*_*sub*_	decryption key of subscriber
*S*_*sub*_	attribute set of subscriber
*m*	plaintext data
*S*_*m*_	Keyword set associated with data m
*C*_*m*_	encrypted data
*T*_*m*_	tags associated with published data
$C_{m}^{\prime }$	pre-decryption ciphertext of data *m*

**Fig 2:**
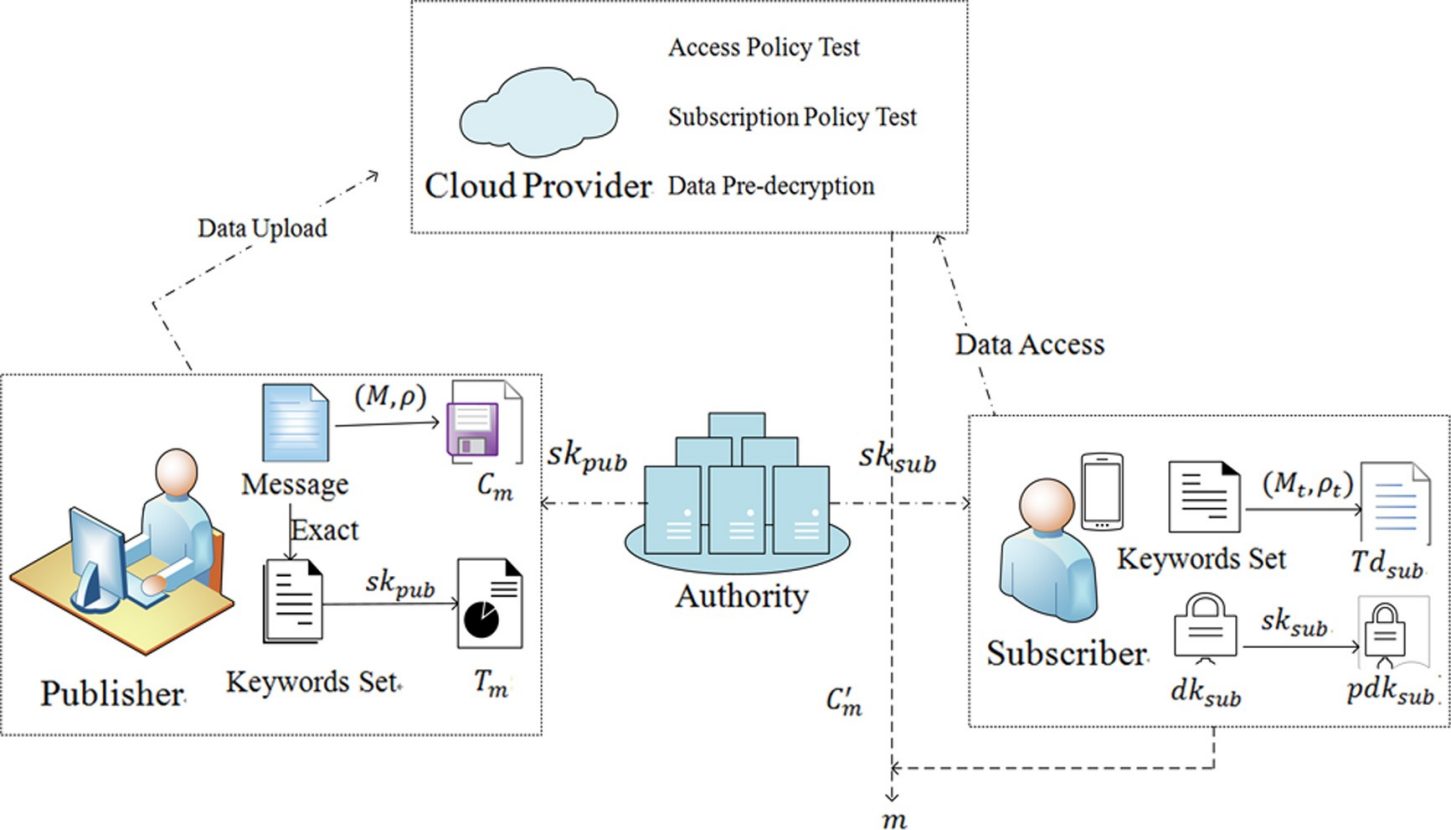
System model of data publish-subscribe service on cloud platforms.

The system model of the data publish-subscribe service on cloud platforms as shown in Fig 2. It consists mainly of four entities: Authority, data publishers, data subscribers, and cloud server. The authority is responsible for establishing the system and generating private keys for data publishers and data subscribers, respectively. The publisher, on the one hand, encrypts data under an access policy about attributes and obtains data ciphertext; on the other hand, encrypts a set of keywords with his/her private key to generate the data tags. The data ciphertext and data tags are then uploaded to the cloud server. The subscriber defines a subscription policy for a set of keywords and uses it to generate search trapdoor, and uses his/her private key to generate the pre-decryption key. The search trapdoor and pre-decryption key are then uploaded to the cloud server. The cloud servers can provide storage and computing services for users because of their considerable storage and computing resources. After receiving the data ciphertext and data tags from the publisher and the search trapdoor and pre-decryption key from the subscriber, the cloud server first conducts the access policy test and the subscription policy test, if and only if the subscriber's attributes satisfy the publisher's access policy and the publisher's tags satisfies the subscriber's subscription policy, the cloud server uses the subscriber's pre-decryption key to pre-decrypt the ciphertext, and sends the pre-decrypted data to the subscriber. The subscriber then decrypts the pre-decrypted data using his/her private key to get the plaintext data.

### D. Security definition of AKPS

**Definition 3** (Td-IND-CKA-Game).Trapdoor indistinguishability security against chosen keyword attacks (Td-IND-CKA) is described in detail in Definition 6 of [1].

**Definition 4** (Tag-IND-CKA-Game). Tag indistinguishability security against chosen keyword attacks (Tag-IND-CKA) is described in detail in Definition 7 of [1].

**Definition 5** (Data-RCCA [11]). The AKPS scheme is Data-RCCA secure if there is no probabilistic polynomial-time adversary who can win in Data-RCCA-Game.

**Definition 6** (Data-RCCA-Game [11]).The Data-RCCA-Game involves a challenger C_Λ_ and an adversary A as follows.

**Setup**: The challenger *C*_Λ_ runs the setup algorithm and gives the public key *pk* to the adversary *A*, but does not divulge master secret key *msk*.

**Phase 1**: The challenger *C*_Λ_ initializes an empty set *D* and an empty table *T* respectively, setting an integer *j* = 0. The adversary *A* makes the following adaptive query to *C*_Λ_.

$Creat(S_{{sub}_{j}}):$ The challenger *C*_Λ_ sets *j*≔*j*+1. It runs the key generation algorithm and pre-decryption algorithm on $S_{{sub}_{j}}$ to obtain the pair $\left({dk}_{{sub}_{j}},{pdk}_{{sub}_{j}}\right)$ and stores the entry $\left(j,S_{{sub}_{j}},{dk}_{{sub}_{j}},\;{pdk}_{{sub}_{j}}\right)$ in table *T*, it then returns to the adversary *A* the pre-decryption key ${pdk}_{{sub}_{j}}$. Note that ${dk}_{{sub}_{j}}$ is decrypt key and ${pdk}_{{sub}_{j}}$ is pre-decrypt key about attribute set *sub*_*j*_.

**Corrupt(***i***)**: If there is an *i*^*th*^ entry $\left(i,S_{{sub}_{i}},{dk}_{{sub}_{i}}{,pdk}_{{sub}_{i}}\right)$ in *T*, then *C*_Λ_ sets $D≔D\cup \{S_{{sub}_{i}}\}$ and returns to the adversary *A* the ${dk}_{{sub}_{i}}$. Otherwise, it returns the symbol ⊥ meaning that there is no such entry.

$Decrypt(i,C_{m}^{\prime }):$ If there is an *i*^*th*^ entry $\left(i,S_{{sub}_{i}},{dk}_{{sub}_{i}}{,pdk}_{{sub}_{i}}\right)$ in *T*, then *C*_Λ_ returns the outcome of decryption to the adversary *A* on takes the tuple ${(dk}_{{sub}_{i}},C_{m}^{\prime })$ as input. If no such entry exists, then it returns ⊥.

**Challenge**: The adversary *A* submits two equal-length messages *m*_0_ and *m*_1_. In addition *A* submits (M*,ρ*) as the challenge access structure, where no queried $S_{{sub}_{i}}\in D$ from phase 1 fulfill it. The challenger *C*_Λ_ then flips a random coin *b*, and encrypts *m*_*b*_ under M*, gives the adversary with $C_{m_{b}}^{*}$.

**Phase 2**: The response is the same as **Phase 1** with the following restrictions:

- *A* cannot conduct a corrupt query that $S_{{sub}_{i}}$ satisfies (M*,ρ*).
- no decryption queries on the message *m*_0_ or *m*_1_.

**Guess**: The adversary *A* outputs a guess *b*′ of *b*.

We define *A*′*s* advantage in Data-RCCA-Game as **Adv**_*data*_ = |Pr[*b*′ = *b*]−1/2|.

**Definition 7** (AKPS Security) The AKPS scheme is secure if it is Td-IND-CKA secure, Tag-IND-CKA secure and Data-RCCA secure.

**Fig 3:**
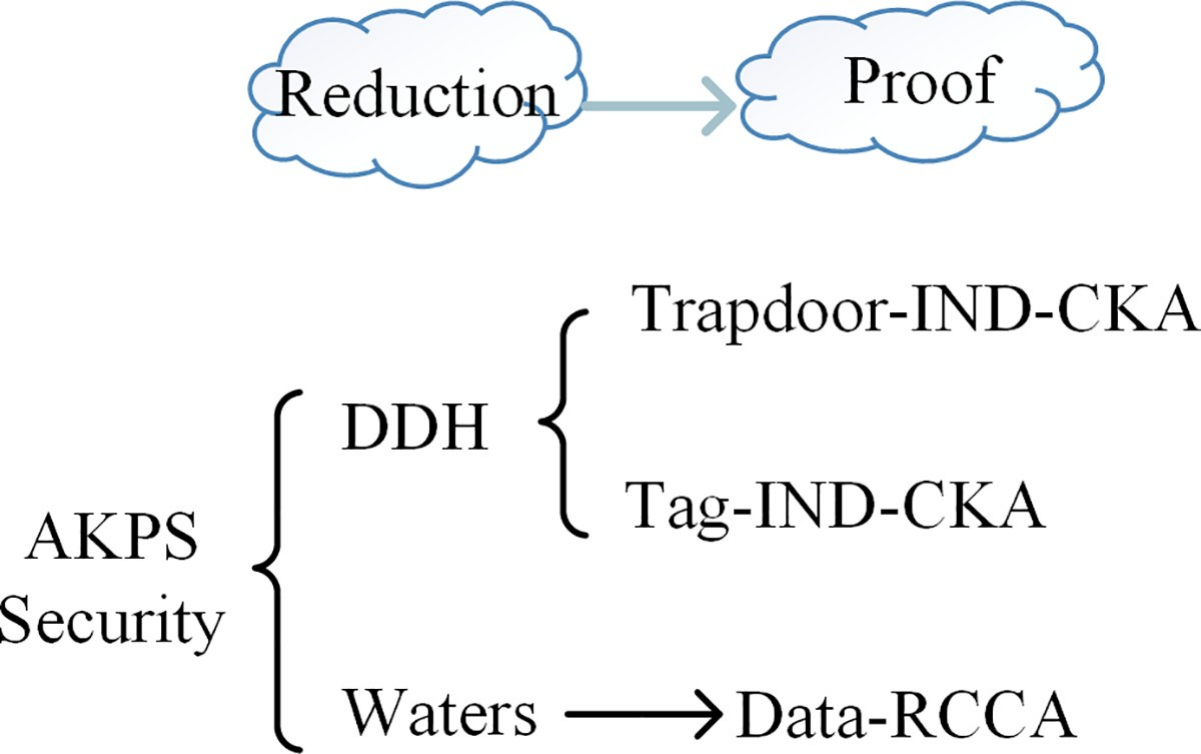
Security proof of the AKPS.

The security of the AKPS scheme (Fig 3) encompasses the following two facets. On the one hand, Td-IND-CKA security and Tag-IND-CKA security can be reduced to the DDH assumption. On the other hand, regarding Data-RCCA security, this can be reduced to the security of Waters scheme [6].

## III. Brief Review of AKPS

We briefly review the AKPS scheme below, which mainly contains five phases: System initialization, Trapdoor generation, Data publication, Policy checking and Pre-decryption, and Data decryption. For a detailed introduction to the scheme, please refer to [1].

**Setup**(1^*k*^)→(*msk*,*pk*). The authority initializes the system by running the setup algorithm. It chooses two multiplicative groups *G* and *G*_*T*_. Let *g* is a generator of *G*. Chooses two hash functions *H*_1_,*H*_2_:{0,1}*→*G* and $\alpha ,\beta ,\gamma ,a\in Z_{p}^{*}$. Then, it sets respectively the master key *msk* and public key *pk* as *msk* = (*g*^*a*^,*α*,*β*,*γ*) and *pk* = (*p*,*g*,*G*, *G*_*T*_,*e*,*e*(*g*,*g*)^*a*^,*g*^*α*^,*g*^*β*^,*g*^*γ*^,*H*_1_,*H*_2_).

**SKeyGen**(*msk*,*pk*,{*S*_*sub*_},{*pub*})→(*sk*_*sub*_,*sk*_*pub*_). For each subscriber *sub* who has the attribute set *S*_*sub*_ and each publisher *pub*, the authority chooses random numbers $r_{sub},r_{pub}\in Z_{p}^{*}$ respectively, and respectively generates the secret key *sk*_*sub*_ and *sk*_*pub*_ for each of subscribers and each of publishers as

$${sk}_{sub}={(K}_{1,sub}=g^{\alpha \beta r_{sub}},K_{2,sub}=g^{\alpha r_{sub}}\cdot g^{\alpha \gamma },K_{3,sub}=g^{a}\cdot g^{\gamma r_{sub}},K_{4,sub}=g^{r_{sub}},\forall att\in S_{sub}:K_{sub,att}={H_{1}(att)}^{r_{sub}}).$$

$${sk}_{pub}={(K}_{1,pub}=g^{\alpha \beta r_{pub}},K_{2,sub}=g^{\beta r_{pub}}\cdot g^{\beta \gamma }).$$

**TdGen**(*sk*_*sub*_,*pk*,(**M**_*t*_,*ρ*_*t*_))→(*Td*_*sub*_,*pkd*_*sub*_,*dk*_*sub*_). To subscribe some interested data, the subscriber first defines an access structure as (**M**_*t*_,*ρ*_*t*_), where **M**_*t*_ is a *n*_*t*_×*l*_*t*_ subscription matrix with *ρ*_*t*_ mapping its rows to keywords. The subscriber first generates a decryption key *dk*_*sub*_ = *z*_*t*_ by selecting a random number $z_{t}\in Z_{p}^{*}$. It then selects $s_{t}\in Z_{p}^{*}$ and a random vector $v_{t}=(s_{t},y_{t,2},\cdots ,y_{t,l})\in Z_{p}^{l_{t}}$, For *j* = 1 to *n*_*t*_, it computes *λ*_*t*,*j*_ = **M**_*t*,*j*_⋅**v**_t_, where **M**_*t*,*j*_ is the vector corresponding to the *j*th row of **M**_*t*_. It then computes *t*_*j*_ = *λ*_*t*,*j*_⋅*z*_*t*_. Using it, subscriber designs the trapdoor as ${Td}_{sub}=(M_{t},{\left\{j,{Td}_{j}\right\}}_{j=1,\cdots ,n_{t}})$

$${Td}_{j}=({Td}_{1,j}={\left(K_{1,sub}∙H_{2}(\rho_{t}(j))\right)}^{t_{j}},{Td}_{2,j}={\left(K_{2,sub}\right)}^{t_{j}},{{Td}_{3,j}=(g^{\alpha })}^{t_{j}},{{Td}_{4,j}=g}^{t_{j}}).$$

In order to protect the keyword leakage from the subscription policy, only **M**_*t*_ of the subscription policy (**M**_*t*_,*ρ*_*t*_) will be sent to the cloud together with the trapdoor, while *ρ*_*t*_ is kept secret against the cloud server. The pre-decryption key *pdk*_*sub*_ is generated as ${pdk}_{sub}=(K_{sub}^{\prime }={\left(K_{3,sub}\right)}^{z_{t}},L_{sub}^{\prime }={\left(K_{4,sub}\right)}^{z_{t}},$ $\forall att\in S_{sub}:K_{sub,att}^{\prime }={\left(g^{\gamma s_{t}}∙K_{sub,att}\right)}^{z_{t}})$.

**Encrypt**(*m*,*S*_*m*_,*pk*,*sk*_*pub*_, (**M**,*ρ*))→(*C*_*m*_,*T*_*m*_). To publish data, the publisher first defines an access policy over attributes of subscribers. The access policy is also described by an LSSS structure as (**M**,*ρ*), where **M** is an *n*×*l* access matrix and *ρ* maps the rows of **M** to attributes. The publisher then runs the following encryption algorithm to encrypt the data, which contains part of the two.

**∙DataEnc**(*m*,*pk*,(**M**,*ρ*))→*C*_*m*_. First, the publisher chooses random values $s_{1},s_{2}\in Z_{p}^{*}$ with two vectors $v_{1}=(s_{1},y_{2}^{\prime },\cdots ,y_{l}^{\prime })$ and $v_{2}=(s_{2},y_{2}^{\prime \prime },\cdots ,y_{l}^{\prime \prime })$. For *i* = 1 to *n*, it computes *λ*_*i*_ = **M**_*i*_⋅**v**_1_ and *μ*_*i*_ = **M**_*i*_⋅**v**_2_, where **M**_*i*_ is the vector corresponding to the *i*th row of **M**. It outputs the ciphertext *C*_*m*_ as

$$C_{m}=((M,\rho ),C=m∙{e(g,g)}^{as_{1}},C^{\prime }=g^{s_{1}},for\;i=1\;to\;n:C_{i}=g^{\gamma \lambda_{1}}∙{H_{1}(\rho (i))}^{-\mu_{i}},D_{i}=g^{\mu_{i}}).$$

**∙TagGen**(*S*_*m*_,*pk*,*sk*_*pub*_,*S*_2_)→*T*_*m*_. The publisher takes the same random number *S*_2_ as input, and outputs the tags as $T_{m}={\left\{W_{i},T_{i}\right\}}_{w_{i}\in S_{m}}$.

$$W_{i}=(W_{1,i}=({K_{1,pub}∙H_{2}(w_{i}))}^{r_{i}},W_{2,i}=({K_{2,pub})}^{r_{i}},W_{3,i}=({g^{\beta })}^{r_{i}},W_{4,i}=g^{r_{i}})$$

$$T_{i}=(T_{1,i}=({K_{1,pub}∙H_{2}(w_{i}))}^{r_{i}^{*}}∙g^{\gamma s_{2}},T_{2,i}=({K_{2,pub})}^{r_{i}^{*}},T_{3,i}=({g^{\beta })}^{r_{i}^{*}},T_{4,i}=g^{r_{i}^{*}})$$

$PolicyTest(C_{m}{,T}_{m},{Td}_{sub},{pdk}_{sub})\to C_{m}^{\prime }\;or⊥.$ The policy test algorithm consists of both access policy test and subscription policy test, and if and only if both policies are satisfied, the algorithm continues to pre-decrypt the data, otherwise it terminates.

**∙Access policy test**. Access policy test is easier than the subscription policy test, because the attributes are not hidden in both the access policy and the pre-decryption key, while the keywords are hidden in both trapdoors and tags. Therefore, algorithm first evaluates whether the attributes of the subscriber can satisfy the access policy associated with data. If the access policy is not satisfied, the policy test algorithm will terminate.

**∙Subscription policy test**. If the access policy is satisfied, it continues to test whether the tags can satisfy the subscription policy in the trapdoor by running the following subroutine:

−**KwdLocate**(*T*_*m*_,*Td*_*sub*_)→*I*_*t*_. Due to the obfuscation of keyword in both the trapdoor and the tags, the algorithm first locates the row number in M_t_ for each tag. When finished search for all the tags, it outputs an index set *I*_*t*_ = {*j*|*ρ*_*t*_(*j*) = *w*_*i*_, ∀*w*_*i*_∈*S*_*m*_}. To test whether the tag *W*_*i*_ and the trapdoor *Td*_*j*_ are corresponding to the same keyword, it checks whether the following equation is equal.

$$\frac{e(W_{1,i},{Td}_{4,j})\cdot e({Td}_{2,j},W_{3,i})}{e({Td}_{1,j},W_{4,i})\cdot e({W_{2,i},Td}_{3,j})}=1$$

∙**Data pre−decryption.** If both access policy and subscription policy are satisfied, the algorithm pre-decrypts the data as follows.

$-PreDecrypt(C_{m},{pdk}_{sub},I_{t})\to C_{m}^{\prime }\;or⊥.$ The cloud server computes *TK*_1_ from the trapdoor and the data tag as

$${TK}_{1}=\prod_{j\in I_{t}}{\left(\frac{e(T_{1,\phi (j)},{Td}_{4,j})\cdot e({Td}_{2,j},T_{3,\phi (j)})}{e({Td}_{1,j},T_{4,\phi (j)})\cdot e(T_{2,\phi (j)},{Td}_{3,j})}\right)}^{c_{t,j}}={e(g,g)}^{\gamma s_{2}s_{t}z_{t}}$$

Similarly, it further computes *TK*_2_ from the ciphertext by using the pre-decryption key as

$${TK}_{2}=\frac{e(C^{\prime },K_{sub}^{\prime })}{\prod_{i\in I}{\left(e(C_{i},L_{sub}^{\prime })\cdot e(D_{i},K_{sub,\rho (i)}^{\prime })\right)}^{c_{i}}}=\frac{{e(g^{s_{1}},g^{a})}^{z_{t}}}{{e(g,g)}^{\gamma s_{2}s_{t}z_{t}}}$$

When obtaining both *TK*_1_ and *TK*_2_, it computes the token as ${{TK=TK}_{1}∙TK}_{2}={e(g,g)}^{as_{1}z_{t}}.$ The pre-decrypted data $C_{m}^{\prime }$ is denoted in an Elgamal encryption form [17] as $C_{m}^{\prime }=(C,TK)=(m∙{e(g,g)}^{as_{1}},{e(g,g)}^{as_{1}}).$

$Decrypt(C_{m}^{\prime },{dk}_{sub})\to m.$ Upon receiving the pre-decrypted data, the data can be easily decrypted by the subscriber as $m=C/{TK}^{{(1/z}_{t})}$.

## IV. Analysis on the security proof of AKPS

In this section, we give a detail analysis about the security proof of the AKPS scheme [1], and point out the flaw of their security proof in [1]. In order to make it clearer, we will name the theorems in the AKPS scheme as Theorem 1, Theorem 2, Theorem 3, and name the security proof of our theorems in section V as Theorem 1', Theorem 2', and Theorem 3'.

The security proof of the AKPS scheme [1] is based on the hardness of the BDH problem, and we find that the security proof of the AKPS scheme is not rigorous and adequate. In the AKPS scheme, Theorem 1 is about the Td-IND-CKA security of the AKPS scheme, it is proved in the random oracle model under the BDH assumption. The BDH assumption and assumption 1 in [1] are as follows.

BDH assumption: Let *A* be an attacker whose running time is polynomial in a security parameter *k*. Given a tuple (*g*,*g*^*a*^,*g*^*b*^,*g*^*c*^), where $a,b,c\in Z_{p}^{*}$. The attacker *A* tries to compute the answer of the BDH problem. We define *A*′*s* advantage in work out the BDH problem as

$${Adv}_{A}^{BDH}(k)=\mathrm{Pr}\left[A(g,g^{a},g^{b},g^{c})={e(g,g)}^{abc}\right].$$

Assumption 1: The BDH problem is said to be computationally intractable if ${Adv}_{A}^{BDH}(k)$ is negligible in *k*.

In their proof, the challenger C interacts with the adversary *A* to conduct the security game. Let’s recall the proof procedure of Theorem 1 about Td-IND-CKA.

Firstly, the challenger C sets master key *msk* = (*g*^*a*^,*α*,*β*,*γ*) and public key *pk* = (*g*,*G*,*G*_*T*_, *g*^*α*^,*g*^*β*^,*g*^*γ*^). Then it generates a private key ${sk}_{sub}=(K_{1,sub}^{*}=g^{\alpha \beta r_{sub}},K_{2,sub}^{*}{=g}^{\alpha r_{sub}}\cdot g^{\alpha \gamma })$ for a subscriber by selecting a random number *r*_*sub*_. Then adversary *A* submits two equal- length keyword vectors $w_{0}^{*}$ and $w_{1}^{*}.$ In addition, *A* also submits a challenge access policy $(M_{t}^{*},\rho_{t}^{*}),$ which can be satisfied by both of the two keyword vectors. C flips a random binary coin *b*∈{0,1}, then selects randomly ${z_{t},s}_{t}\in Z_{p}^{*}$ and computes share component $\lambda_{t,j}=M_{t,j}^{*}\cdot v$ and *t*_*j*_ = *λ*_*t*,*j*_⋅*z*_*t*_. Using generated private key *sk*_*sub*_, C generates the challenge trapdoor ${Td}_{sub}^{\left(b\right)}$ as

$${Td}_{j}^{\left(b\right)}=({Td}_{1,j}^{\left(b\right)}={\left(K_{1,sub}^{*}∙H_{2}(w_{b,j})\right)}^{t_{j}},{Td}_{2,j}^{\left(b\right)}={\left(K_{2,sub}^{*}\right)}^{t_{j}},{{Td}_{3,j}^{\left(b\right)}=(g^{\alpha })}^{t_{j}},{{Td}_{4,j}^{\left(b\right)}=(g)}^{t_{j}}).$$

In the challenge phase, the challenger C conducts the simulation of trapdoor corresponding the keyword vectors are submitted by the adversary *A*. Subsequently, the adversary *A* needs to compute ${e(g,g)}^{\beta \gamma \alpha t_{j}}$ as the solution to the BDH problem, where given the BDH tuple as $g^{\beta },g^{\gamma },g^{c}=g^{\alpha t_{j}}$.

After challenger C gives the challenge trapdoor ${Td}_{sub}^{\left(b\right)}$ and the input of BDH problem $g^{\beta },g^{\gamma },g^{c}=g^{\alpha t_{j}}$ to adversary *A*, the adversary *A* needs to calculate the solution of the BDH problem ${e(g,g)}^{\beta \gamma \alpha t_{j}}$ as:

$${e(g,g)}^{\beta \gamma \alpha t_{j}}=\frac{e({Td}_{2,j}^{\left(b\right)},\;g^{\beta })\cdot e(H(w_{b^{\prime },j}),{Td}_{4,j}^{\left(b\right)})}{e({Td}_{1,j}^{\left(b\right)},g)}=e(g^{\alpha \gamma t_{j}},g^{\beta })∙\frac{e(H(w_{b^{\prime },j}),g^{t_{j}})}{e(H(w_{b,j}),g^{t_{j}})}$$

The adversary *A* then outputs a random guess *b*′ of *b*, if *b*′ = *b*, it means that the adversary *A* calculates the solution of the BDH problem. Since it is a random guess, the guessing probability of this case is 1/2. If *b*′ ≠ *b*, it means that the adversary *A* does not calculate the solution of the BDH problem, and the probability of this case is also 1/2. Therefore, the adversary *A* can solve the BDH problem with a probability of 1/2, but did not attack the real AKPS scheme. In other words, the adversary *A* has no advantage in the attack of the scheme and it does not need to interact with C. The only thing that needs the adversary *A* to do is just a random guess *b*′ of *b*. From the above analysis, we can obtain the conclusion that the security proof of the AKPS scheme is not adequate.

Similarly, it’s the same as theorem 1 in theorem 2, which is about the security of the Tag-IND-CKA.

The theorem 3 of the AKPS scheme [1] is that the AKPS is Data-CPA secure in the random oracle if the decision *q*-parallel BDHE assumption holds. In Theorem 3, the outsourcing decryption technology of the AKPS scheme is based on the technique in [11]. Specifically, to enable the cloud server to pre-decrypt the data, the pre-decryption key generation algorithm is constructed by employing the technique from [11], which is proven to be semantic security against chosen plaintext attacks by using literature [6]. Similarly, the AKPS scheme can be proven to be Data-CPA secure, nevertheless the original AKPS scheme [1] dose not gives specific proof. Through researching the literature [11], we conclude that the security proof in [11] is reduced to Waters scheme [6], which is proven to be Data-RCCA secure. Therefore, using the proof method in [6], we demonstrate Data-RCCA security on the AKPS scheme, and the specific proof can be found in Theorem 3' of Part V.

## V. Improving the security Proof of AKPS

In this section, we will give our new security proof of the AKPS scheme about the security of the trapdoor and tag. In addition, we give a new security proof of the Data-RCCA security for the AKPS scheme.

Loosely speaking, the security proof of a cryptographic scheme can be described as follows. A pre-defined difficult problem is deployed by the challenger, and the adversary is supposed to attack the constructed scheme in a security game. The challenger interacts with the adversary to conduct the security game, and inserts the difficulty problem into the scheme. Only if the adversary attacks the scheme successfully with a non-negligible advantage, then the challenger can figure out the difficulty problem correctly with a non-negligible advantage. So if the difficulty problem is really hard to solve then the constructed scheme is secure. Our security proof of the AKPS scheme is based on the DDH assumption in this section. The new security proofs are given as theorem 1' and theorem 2', where theorem 1' is about Td-IND-CKA secure, and theorem 2' is about Tag-IND-CKA secure. While theorem 3' is about Data-RCCA secure which is a complete new proof for the security of AKPS scheme [1]. Our security proof is different from the original one in [1], one of the main differences is that our proof is under the DDH assumption while their proof is BDH assumption, as we cannot apply the BDH assumption to the security proof of the AKPS scheme [1].

### Td-IND-CKA Security

**Theorem 1'.**The AKPS scheme is Td-IND-CKA secure in the random oracle model if the DDH problem is intractable.

**Proof**. Suppose that there is an adversary *A*_1_ with non-negligible advantage ε_1_ in the Td-INK-CKA Game against the construction of AKPS scheme in [1]. We build a simulator *S*_1_ that can figure out the DDH problem with advantage ε_1_/2, the simulation proceeds as follows. Let the challenger *C*_1_ generates public parameter (*e*,*g*,*G*,*G*_*T*_) and *G* = <*g*>. *C*_1_ flips a fair binary coin *φ*∈{0,1} outside of $S_{1}^{\prime }s$ view. if *φ* = 1, *C*_1_ sets (*A*,*B*,*Z*) = (*g*^*x*^,*g*^*y*^,*g*^*xy*^), else it sets (*A*,*B*,*Z*) = (*g*^*x*^,*g*^*y*^,*g*^*z*^), where given the values *x*,*y*,*z* are chosen randomly from $Z_{p}^{*}.$

**Setup:** The simulator *S*_1_ runs **Setup**(*k*) algorithm, sets *msk* = (*α*,*β*,*γ*) for value *β* had known by *S*_1_ that chosen randomly from $Z_{p}^{*}$, where implicitly sets *α* = *x*,*γ* = *y*.Then *S*_1_ computes public key *pk* = (*g*^*α*^,*g*^*β*^,*g*^*γ*^) and sends it to *A*_1_. In addition, *S*_1_ generates respectively private key *sk*_*pub*_ and *sk*_*sub*_ for publisher and subscriber as

$${sk}_{pub}=(K_{1,pub}^{*}=g^{\alpha \beta r_{pub}}=A^{\beta r_{pub}},K_{2,pub}^{*}=g^{\beta r_{pub}}\cdot g^{\beta \gamma }=g^{\beta r_{pub}}\cdot B^{\beta })$$

$${sk}_{sub}=(K_{1,sub}^{*}=g^{\alpha \beta r_{sub}}=A^{\beta r_{sub}},K_{2,sub}^{*}=g^{\alpha r_{sub}}\cdot g^{\alpha \gamma }=A^{r_{sub}}\cdot Z)$$

Each of which does not be divulged to *A*_1_, where $r_{pub},r_{sub}{\in Z}_{p}^{*}$.

**Phase 1:** The *A*_1_ can adaptively query, *S*_1_ answers queries as following.

*H*_2_ **query**. *A*_1_ can query the random oracle *H*_2_. To respond to *H*_2_ queries, *S*_1_ maintains a list of tuple $\left(w_{i},g^{\tau_{i}}\right)$ called the *H*_2_ *list*, which is initially empty. When *A*_1_ queries *H*_2_ at a specific point *w*_*i*_∈{0,1}*, *S*_1_ responds as follows:

—If the query *w*_*i*_ already appears on the *H*_2_ *list* in a tuple $\left(w_{i},g^{\tau_{i}}\right)$, then *S*_1_ responds *A*_1_ with the tuple ${H_{2}(w_{i})=g}^{\tau_{i}}$.

—Else, *S*_1_ chooses a random value $\tau_{i}{\in Z}_{p}^{*}$, sets ${H_{2}(w_{i})=g}^{\tau_{i}}$ and stores the tuple $\left(w_{i},g^{\tau_{i}}\right)$ into *H*_2_ *list*.

Tag query. *A*_1_ is allowed to issue queries for the tag of a set of keywords *kw* = (*w*_1_,⋯,*w*_*n*_), *S*_1_ chooses randomly $s_{2},r_{i},r_{i}^{*}{\in Z}_{p}^{*}$. For each keyword *w*_*i*_, using the publisher’s private key *sk*_*pub*_, *S*_1_ makes the corresponding response $T^{\prime }={\left\{W_{i},T_{i}\right\}}_{w_{i}\in KW}$ as

$$W_{i}=(W_{1,i}=({K_{1,pub}^{*}∙H_{2}(w_{i}))}^{r_{i}}=({A^{\beta r_{pub}}∙g^{\tau_{i}})}^{r_{i}},W_{2,i}=({K_{2,pub}^{*})}^{r_{i}}=({g^{\beta r_{pub}}\cdot B^{\beta })}^{r_{i}},W_{3,i}=({g^{\beta })}^{r_{i}},W_{4,i}=g^{r_{i}})$$

$$T_{i}=(T_{1,i}=({K_{1,pub}^{*}∙H_{2}(w_{i}))}^{r_{i}^{*}}∙g^{\gamma s_{2}}=({A^{\beta r_{pub}}∙H_{2}(w_{i}))}^{r_{i}^{*}}∙B^{s_{2}},T_{2,i}=({K_{2,pub}^{*})}^{r_{i}^{*}}=({g^{\beta r_{pub}}\cdot B^{\beta })}^{r_{i}^{*}},T_{3,i}=({g^{\beta })}^{r_{i}^{*}},T_{4,i}=g^{r_{i}^{*}})$$

**Challenge**: Let two equal-length keyword vectors $w_{0}=(w_{0,1},\cdots ,w_{0,n^{*}})$, $w_{1}=(w_{1,1},\cdots ,w_{1,n^{*}})$ submitted by the adversary *A*_1_, which have not been queried in above phase. In addition, *A*_1_ submits a challenge access policy $\left(M_{t}^{*},\rho_{t}^{*}\right)$, which can be satisfied by both of the two vectors. *S*_1_ flips a random binary coin *b*∈{0,1}, selects randomly ${z_{t},s}_{t}\in Z_{p}^{*}$ and a vector $v=(s_{t},y_{2}^{\prime \prime \prime },\cdots ,y_{n}^{\prime \prime \prime })$. Subsequently, it computes $\lambda_{t,j}=M_{t,j}^{*}\cdot v$ and *t*_*j*_ = *λ*_*t*,*j*_∙*z*_*t*_. Using subscriber’s private key *sk*_*sub*_, *S*_1_ generates the challenge trapdoor ${Td}_{sub}^{\left(b\right)}$ as

$${Td}_{sub}^{\left(b\right)}=(M_{t}^{*},{\left\{j,{Td}_{j}^{\left(b\right)}\right\}}_{j=1,\cdots ,n^{*}})$$

$${Td}_{j}^{\left(b\right)}=({Td}_{1,j}^{\left(b\right)}={\left(K_{1,sub}^{*}∙H_{2}(w_{b,j})\right)}^{t_{j}}={\left(A^{\beta r_{sub}}∙g^{\tau_{i}}\right)}^{t_{j}},{Td}_{2,j}^{\left(b\right)}={\left(K_{2,sub}^{*}\right)}^{t_{j}}={\left(A^{r_{sub}}\cdot Z\right)}^{t_{j}},{{Td}_{3,j}^{\left(b\right)}=(g^{\alpha })}^{t_{j}}=A^{t_{j}},{{Td}_{4,j}^{\left(b\right)}=g}^{t_{j}}.$$

${Td}_{2,j}^{\left(b\right)}$ is the correct trapdoor component only if *Z* = *g*^*xy*^, else ${Td}_{2,j}^{\left(b\right)}$ is a random element.

**Phase 2**: It’s the same as **Phase 1**.

**Guess**: *A*_1_ outputs a guess *b*′ of *b*. if *b*′ = *b*, then *S*_1_ outputs *φ* = 1 to indicate that it is given a valid DDH tuple, otherwise it outputs *φ* = 0 to indicate that it is a random element. The advantage of *S*_1_ to solve DDH problem is

$$\frac{1}{2}\times \mathrm{Pr}\left[\varphi^{\prime }=\varphi |\varphi =1\right]+\frac{1}{2}\times \mathrm{Pr}\left[\varphi^{\prime }=\varphi |\varphi =0\right]-\frac{1}{2}$$

$$=\frac{1}{2}\times \left(\frac{1}{2}+\varepsilon_{1}\right)+\frac{1}{2}∙\frac{1}{2}-\frac{1}{2}=\frac{\varepsilon_{1}}{2}$$

Therefore, if the *A*_1_ has a non-negligible advantage *ε*_1_ in the above game, then we can build a simulator *S*_1_ which can break the DDH problem with non-negligible advantage *ε*_1_/2, which is an intractable problem. Hence, the theorem 1.

### B.Tag-IND-CKA Security

**Theorem 2'.**The AKPS scheme is Tag-IND-CKA secure in the random oracle model if the DDH problem is intractable.

**Proof**. Suppose that the adversary *A*_2_ with non-negligible advantage *ε*_2_ in the Tag-INK-CKA Game against the construction of AKPS scheme in [1]. We build a simulator *S*_2_ that can solve the DDH problem with advantage *ε*_2_/2. The simulation proceeds as follows. Let the challenger *C*_2_ generates the parameter (*e*,*g*,*G*,*G*_*T*_) and *G* = <*g*>. Then it flips a fair binary coin *ϕ*∈{0,1}, outside of $S_{2}^{\prime }s$ view. If *ϕ* = 1, *C*_2_ sets (*A*,*B*,*Z*) = (*g*^*x*^,*g*^*y*^,*g*^*xy*^), otherwise it sets (*A*,*B*,*Z*) = (*g*^*x*^,*g*^*y*^,*g*^*z*^) for values *x*,*y*,*z* chosen randomly from $Z_{p}^{*}$.

**Setup**: The simulator *S*_2_ runs **Setup**(*k*) algorithm, sets *msk* = (*α*,*β*,*γ*) for value *α* had known by *S*_2_ chosen randomly from $Z_{p}^{*}$, where implicitly sets (*β* = *x*,*γ* = *y*). Then *S*_2_ computes public key *pk* = (*g*^*α*^,*g*^*β*^,*g*^*γ*^) and sends it to *A*_2_. In addition, *S*_2_ generates respectively private key *sk*_*pub*_ and *sk*_*sub*_ for publisher and subscriber as

$${sk}_{pub}=(K_{1,pub}^{*}=g^{\alpha \beta r_{pub}}=A^{\alpha r_{pub}},K_{2,pub}^{*}=g^{\beta r_{pub}}\cdot g^{\beta \gamma }=A^{r_{pub}}\cdot Z)$$

$${sk}_{sub}=(K_{1,sub}^{*}=g^{\alpha \beta r_{sub}}=A^{\alpha r_{sub}},K_{2,sub}^{*}=g^{\alpha r_{sub}}\cdot g^{\alpha \gamma }=g^{{\alpha r}_{sub}}\cdot B^{\alpha })$$

Each of which does not be divulged to *A*_2_, where $r_{pub},r_{sub}{\in Z}_{p}^{*}$.

**Phase 1**: The adversary *A*_2_ can adaptively query, *S*_2_ answers queries as following.

*H*_2_ **query**. *A*_2_ can query the random oracle *H*_2_. To respond to *H*_2_ queries, *S*_2_ maintains a list of tuple $\left(w_{i},g^{\tau_{i}}\right)$ called the *H*_2_ *list*, which is initially empty. When *A*_2_ queries *H*_2_ at a specific point *w*_*i*_∈{0,1}*, *S*_2_ responds as follows:

—If the query *w*_*i*_ already appears on the *H*_2_ *list* in a tuple $\left(w_{i},g^{\tau_{i}}\right)$, then *S*_2_ responds *A*_2_ with the tuple ${H_{2}(w_{i})=g}^{\tau_{i}}$.

—Else, *S*_2_ chooses a random value $\tau_{i}{\in Z}_{p}^{*}$, sets ${H_{2}(w_{i})=g}^{\tau_{i}}$ and stores the tuple $\left(w_{i},g^{\tau_{i}}\right)$ into *H*_2_ *list*.

**Trapdoor query**: *A*_2_ is allowed to issue queries for the trapdoor of a set of keywords *kw*′ and a subscription policy *SP*_*kw*′_ (the subscription policy is described as $\left(M_{t}^{\prime },\rho_{t}^{\prime }\right)$) constructed over *kw*′.

*S*_2_ chooses randomly ${z_{t}}^{\prime },{s_{t}}^{\prime }\in Z_{p}^{*}$, sets vector $v^{\prime }=({s_{t}}^{\prime },y_{2}^{\circ },\cdots ,y_{n}^{\circ })$ and computes $\lambda_{t,j}^{\prime }=M_{t}^{\prime }∙v^{\prime }$ and $t_{j}^{\prime }=\lambda_{t,j}^{\prime }{{∙z}_{t}}^{\prime }$. Then, using subscriber’s private key *sk*_*sub*_, *S*_2_ generates the corresponding challenge trapdoor ${Td}_{sub}^{\prime }$ as

$${Td}_{sub}^{\prime }=(M_{t}^{\prime },{\left\{j,{Td}_{j}^{\prime }\right\}}_{j=1,\cdots ,n^{*}})$$

$${Td}_{j}^{\prime }=({Td}_{1,j}^{\prime }={\left(K_{1,sub}^{*}∙H_{2}(\rho_{t}(j))\right)}^{t_{j}^{\prime }}={\left(A^{{\alpha r}_{sub}}∙g^{\tau_{\rho_{t}(j)}}\right)}^{t_{j}^{\prime }},{Td}_{2,j}^{\prime }={\left(K_{2,sub}^{*}\right)}^{t_{j}^{\prime }}={\left(g^{{\alpha r}_{sub}}\cdot B^{\alpha }\right)}^{t_{j}^{\prime }},{{Td}_{3,j}^{\prime }=(g^{\alpha })}^{t_{j}^{\prime }},{{Td}_{4,j}^{\prime }=g}^{t_{j}^{\prime }}.$$

**Challenge**: Let **kw**_**0**_ = (*w*_0,1_,⋯,*w*_0,*n*_), **kw**_**1**_ = (*w*_1,1_,⋯,*w*_1,*n*_) are two equal-length keyword vectors submitted by the adversary *A*_2_,which have not been queried in above phase. Then *S*_2_ flips a random coin *b*∈{0,1} and selects randomly $s_{2},r_{i},r_{i}^{*}{\in Z}_{p}^{*}$. Using publisher’s private key *sk*_*pub*_, *S*_2_ generates the challenge tag $T^{\left(b\right)}={\left\{W_{i}^{\left(b\right)},T_{i}^{\left(b\right)}\right\}}_{w_{b,i}\in {KW}_{b}}$ as

$$W_{i}^{\left(b\right)}=(W_{1,i}^{\left(b\right)}=({K_{1,pub}^{*}∙H_{2}(w_{b,i}))}^{r_{i}}=({A^{\alpha r_{pub}}∙g^{\tau_{b,i}})}^{r_{i}},W_{2,i}^{\left(b\right)}=({K_{2,pub}^{*})}^{r_{i}}=({A^{r_{pub}}\cdot Z)}^{r_{i}},W_{3,i}^{\left(b\right)}=({g^{\beta })}^{r_{i}}=A^{r_{i}},W_{4,i}^{\left(b\right)}=g^{r_{i}})$$

$$T_{i}^{\left(b\right)}=(T_{1,i}^{\left(b\right)}=({K_{1,pub}^{*}∙H_{2}(w_{i}))}^{r_{i}^{*}}∙g^{\gamma s_{2}}=({A^{\alpha r_{pub}}∙g^{\tau_{b,i}})}^{r_{i}^{*}}∙B^{s_{2}},T_{2,i}^{\left(b\right)}=({K_{2,pub}^{*})}^{r_{i}^{*}}=({A^{r_{pub}}\cdot Z)}^{r_{i}^{*}},T_{3,i}^{\left(b\right)}=({g^{\beta })}^{r_{i}^{*}}=A^{r_{i}^{*}},T_{4,i}^{\left(b\right)}=g^{r_{i}^{*}})$$

$W_{2,i}^{\left(b\right)}$ and $T_{2,i}^{\left(b\right)}$ are the correct trapdoor component only if *Z* = *g*^*xy*^, else the component of both are random element.

**Phase 2**: It’s the same as **Phase 1**.

**Guess**: *A*_2_ outputs a guess *b*′ of *b*. if *b*′ = *b*,then *S*_2_ outputs *ϕ* = 1 to indicate that it is given a valid DDH tuple, else it outputs *ϕ* = 0 to indicate that it is a random element. The advantage of *S*_2_ to solve the DDH problem is

$$\frac{1}{2}\times \mathrm{Pr}\left[\phi^{\prime }=\phi |\phi =1\right]+\frac{1}{2}\times \mathrm{Pr}\left[\phi^{\prime }=\phi |\phi =0\right]-\frac{1}{2}$$

$$=\frac{1}{2}\times \left(\frac{1}{2}+\varepsilon_{2}\right)+\frac{1}{2}∙\frac{1}{2}-\frac{1}{2}=\frac{\varepsilon_{2}}{2}$$

Therefore, if the *A*_2_ has a non-negligible advantage *ε*_2_ in the above game then we can build a simulator *S*_2_ which can break the DDH problem with non-negligible advantage *ε*_2_/2, which is an intractable problem. Hence, the theorem 2.

### C.Data-RCCA Security

**Theorem 3'**. The AKPS scheme is RCCA secure in random oracle model assuming that the Waters scheme [6] is a selectively CPA-secure scheme.

**Proof**. Suppose there is a polynomial-time adversary *A*_3_ that can attack our scheme in the selective RCCA security model with advantage *ε*_3_. we build a simulator *S*_3_ that can attack the Waters scheme in the selective CPA-security model with advantage *ε*_3_ minus a negligible amount. Let *C*_3_ be the challenger of the Waters scheme.

**Init**: The simulator *S*_3_ runs *A*_3_. *A*_3_ chooses the challenge access structure (M*,ρ*), which *S*_3_ sends it to the Waters challenger *C*_3_.

**Setup**: *S*_3_ queries *C*_3_ to obtain the Waters public key *pk* = (*g*,*e*(*g*,*g*)^*a*^,*g*^*α*^) and a hash function *H*_1_. It sends these to *A*_3_ as the public parameters.

**Phase 1**: The simulator *S*_3_ initializes an empty table *T*, an empty set *D* and an integer *j* = 0. Then *S*_3_ responses to *A*_3_ as follows:

$Creat(S_{{sub}_{j}}):\;S_{3}$ sets *j*≔*j*+1. It has the condition of the two.

—If (M*,ρ*) be satisfied by $S_{{sub}_{j}},$ then it choose a “fake” pre-decryption key as follows. It chooses $d\in Z_{p}^{*}$ randomly and sends $S_{{sub}_{j}}$ to *C*_3_ to query the corresponding user secret key ${sk}_{{sub}_{j}}$, then set ${pdk}_{{sub}_{j}}={sk}_{{sub}_{j}}$ and implicitly set ${dk}_{{sub}_{j}}=d.$ where secret key pairs ${(dk}_{{sub}_{j}},{pdk}_{{sub}_{j}})$ is incomplete, but that ${pdk}_{{sub}_{j}}$ is be fittingly distributed if *d* was substitute for the unknown value *z*_*t*_ = *d*/*a*.

—Otherwise, *S*_3_ sends $S_{{sub}_{j}}$ to *C*_3_ to request the corresponding secret key, and *C*_3_ replies with the secret key ${sk}_{{sub}_{j}}=(pk,K_{3,{sub}_{j}},K_{4,{sub}_{j}},\{K_{{sub}_{j},att}{\}}_{att\in S_{{sub}_{j}}}).$ Note that we substitute the symbol of Waters scheme for the symbol of AKPS scheme, which represent the same meaning with it. The algorithm chooses random value ${z_{t},s}_{t}\in Z_{p}^{*}$ where ${dk}_{{sub}_{j}}=z_{t}$, and sets pre-decryption key ${pdk}_{{sub}_{j}}$ as ${pdk}_{{sub}_{j}}=(K_{{sub}_{j}}^{\prime }={\left(K_{3,{sub}_{j}}\right)}^{z_{t}},L_{{sub}_{j}}^{\prime }={\left(K_{4,{sub}_{j}}\right)}^{z_{t}},K_{{sub}_{j},att}={\left(g^{\gamma s_{t}}∙K_{sub,att}\right)}^{z_{t}}:\forall att\in S_{sub})$.

Finally, store $\left(j,S_{{sub}_{j}},{dk}_{{sub}_{j}}{,pdk}_{{sub}_{j}}\right)$ in table *T* and return ${pdk}_{{sub}_{j}}$ to *A*_3_.

**Corrupt**(*i*): *A*_3_ can adaptively queries any secret corresponding to the access structure expect (M*,ρ*). If there is an *i*^*th*^ entry $\left(i,S_{{sub}_{i}},{dk}_{{sub}_{i}}{,pdk}_{{sub}_{i}}\right)$ in table *T*, thent *S*_3_ sets *D* as $D≔D\cup \{S_{{sub}_{i}}\}.$ It then returns to the adversary *A*_3_ with the ${dk}_{{sub}_{i}}$, or ⊥ otherwise.

$Decrypt(i,C_{m}^{\prime }):$ Thinking that *S*_3_ and *A*_3_ can obtain the *pdk*_*sub*_ values for all keys created, either can realize the pre-decryption algorithm. Therefore, we assume that ciphertexts which we obtain are already partially decrypted. Let *CT* = (*C*,*TK*) be associated with structure (**M**_*χ*_,*ρ*_*χ*_). Extract the entry $\left(i,S_{{sub}_{i}},{dk}_{{sub}_{i}}{,pdk}_{{sub}_{i}}\right)$ from table *T*. If it is not exist there or $S_{{sub}_{i}}$ unsatisfied (**M**_*χ*_,*ρ*_*χ*_), return ⊥ to *A*_3_.

—If *i*^*th*^ does not satisfy the (M*,ρ*), obtain the records $\left(z_{t},{pdk}_{{sub}_{j}}\right)$ from table *T*, then parse it to output $m=C/{TK}^{{(1/z}_{t})}$ in response of *A*_3_ queries. If none exist, return ⊥ to *A*_3_.

—If *i*^*th*^ satisfy the policy (M*,ρ*), obtain the records $\left(d,{pdk}_{{sub}_{j}}\right)$ from table *T*, then parse it to output $m=C/{TK}^{{(1/z}_{t})}$ in response of *A*_3_ queries. If none exist, return ⊥ to *A*_3_.

**Challenge**: *A*_3_ submits two equal-length messages *m*_0_ and *m*_1_, *S*_3_ sends it to challenger *C*_3_, then the challenger *C*_3_ flips a random coin *b*∈{0,1} to obtain the challenge ciphertext $C_{m_{b}}^{*}=(C,C^{\prime },{{\{C}_{i},D_{i}\}}_{i\in [1,n]})$ under the access structure (M*,ρ*), then *S*_3_ sends the $C_{m_{b}}^{*}$ to *A*_3_.

**Phase 2**: The response is the same as **Phase 1**, expect that no decryption queries would be either *m*_0_ or *m*_1_.

**Guess**: Eventually, *A*_3_ outputs a guess *b*′∈{0,1}, then *S*_3_ outputs *b*′.

Hence, if the adversary *A*_3_ can break the AKPS scheme with the given advantage *ε*_3_, then *S*_3_ can break the Waters scheme with the same advantage. Hence, the theorem 3.

## VI. Conclusion

We analyze the security proof of AKPS scheme [1] for indistinguishability of tag and trapdoor and show that the security proof of the AKPS scheme is not rigorous and adequate, although the construction of AKPS scheme is remarkable. Based on it, we give an improving security proof of AKPS scheme for its Tag-IND-CKA security and Td-IND-CKA security based on the DDH assumption. Furthermore, by using of the conclusion that the Waters scheme in [6] is selectively CPA-secure, we manifest that the AKPS scheme realizes data replayable secure against replayable chosen ciphertext attack (RCCA), which has a higher level of security than the security of the indistinguishability of the Data-CPA in original AKPS scheme, which is mentioned but not demonstrated. Moreover, there are a number of issues that need to be studied and solved for the attribute-keyword based data publish-subscribe scheme on the cloud platforms. Firstly, new AKPS scheme should be designed to cope with the situation that a subscription policy is spelled with mistakes of interesting words, for example, 'compute' may be spelled as 'compote' or 'compue'. Secondly, for the situations where the subscriber's attributes may have been changed, such as revoke, update, increase etc, how to design efficient attribute revocation and update algorithm to realize the dynamic management of attributes, and protect the forward and backward security of the algorithm is a promising study topics. Thirdly, in publish-subscribe system, how to add the concept of time into the access policy to avoid illegal data access in the case of private key is leaked. These three aspects will be the focus of our future work.

## Supporting information

S1 File Computational cost in the AKPS scheme. (DOCX)

S2 File The runtime of cryptographic operations. (DOCX)
