# Supplementary material for: Improving the proof of “Privacy-preserving attribute-keyword based data publish-subscribe service on cloud platforms”
Source: PLoS One. 2019 Feb 25;14(2):e0212761. doi: 10.1371/journal.pone.0212761 (PMC6388936; doi:10.1371/journal.pone.0212761)
Supplement: S1 File — (DOCX) [file pone.0212761.s001.docx]

**S1 File**

The computational cost of each component in the AKPS scheme is as follows:

**Computational cost in the AKPS scheme**

| component | TAKPS |
| --- | --- |
| $pk$ | 3E^1^+P^2^ |
| ${sk}_{sub}$ | 2M^3^ + (5+$u_{1}$^4^)E |
| ${sk}_{pub}$ | M+3E |
| ${Td}_{sub}$ | 4$k_{1}$^5^E+$k_{1}$M |
| $C_{m}$ | (2+3$u_{2}$^6^)E+(1+$u_{2}$)M |
| $T_{m}$ | (1+8$k_{2}$^7^)E+2$k_{2}$M |
| $c_{m}^{'}$ | (2$u_{3}$^8^+$k_{3}$^9^)E+2M+2P |

^1^E: exponentiation in $G$.

^2^P: pairing operation.

^3^M: multiplication in $G$.

^4^$u_{1}$: the number of subscriber’s attribute.

^5^$k_{1}$: the number of keyword in subscription policy;

^6^$u_{2}$: the number of attribute in access policy.

^7^$k_{2}$: the number of keyword in tags;

^8^$u_{3}$: the number of the attribute of subscriber satisfying access policy.

^9^$k_{3}$: the number of the keyword of tags satisfying subscription policy.
