# Supplementary material for: Improving the proof of “Privacy-preserving attribute-keyword based data publish-subscribe service on cloud platforms”
Source: PLoS One. 2019 Feb 25;14(2):e0212761. doi: 10.1371/journal.pone.0212761 (PMC6388936; doi:10.1371/journal.pone.0212761)
Supplement: S2 File — (DOCX) [file pone.0212761.s002.docx]

**S2 File**

The runtime of the AKPS scheme refers to Pairing Based Cryptography (PBC) library. The specific operation is as follows:

**The runtime of cryptographic operations**

| Operating | Ad | Ne | M | In | Ex | Add | Neg | PM | Mul | Inv | E' | Exp | P |
| --- | --- | --- | --- | --- | --- | --- | --- | --- | --- | --- | --- | --- | --- |
| Times/ms | 0.001 | 0.000 | 0.001 | 0.004 | 0.067 | 0.038 | 0.001 | 8.006 | 0.013 | 0.041 | 1.882 | 1.882 | 16.064 |

^1^Ad: an addition operation in ;

^2^Ne: the inverse in addition operation in ;

^3^M: a multiplication operation in ;

^4^In: the inverse in multiplication operation in ;

^5^Ex: an exponentiation operation in ;

^6^Add: an addition operation in ;

^7^Neg: the inverse in addition operation in ;

^8^PM: a point multiplication operation in ;

^9^Mul: a multiplication operation in ;

^10^Inv: the inverse in multiplication operation in ;

^11^E': an exponentiation operation in ;

^12^Exp: an exponentiation operation in ;

^13^P: an bilinear pairings operation in .

We mainly consider three kinds of operations on time complexity: exponential operation, multiplication operation and pair operation.
